# Supplementary material for: Content-rich biological network constructed by mining PubMed abstracts
Source: BMC Bioinformatics. 2004 Oct 8;5:147. doi: 10.1186/1471-2105-5-147 (PMC528731; doi:10.1186/1471-2105-5-147)
Supplement: Additional File 2 — The original results of the above study (non-essential files are deleted to keep the file size under the limit set by BMC bioinformatics). [file 1471-2105-5-147-S2.bz2 › chilibotAdditionalFile2/dip50/342ID8409434E4302/html/IL7_IL7R.html]

 


 **IL7** and **IL7R** 
  
Found 103 abstracts in PubMed, retrieved 50.  
 

 What does Google say? 
 PDF only 
| .edu only 

---

**Interactive relationship** (e.g. stimulation, inhibition, etc)

**Neutral relationship**- This can be explained by the finding that the receptor complexes for TSLP and IL 7  [ **IL7** ]  both contain the IL 7R alpha  [ **IL7R** ]  chain.  Ref: 10570284 J Immunol, 1999
- The cellular basis underlying this dichotomy was revealed by the finding that the fetal IL 7R alpha  [ **IL7R** ]  population responded equally well to IL 7  [ **IL7** ]  and RANKL to express LT alpha beta.  Ref: 12479827 Immunity, 2002
- B lineage potential was largely associated with interleukin 7  [ **IL7** ]  receptor alpha IL 7R alpha  [ **IL7R** ]  expression and was selectively driven in culture by IL 7  [ **IL7** ] .  Ref: 12393656 Blood, 2002
- In humans, although both T cell and B cell progenitors express the functional IL 7  [ **IL7** ]  receptor that consists of IL 7R alpha  [ **IL7R** ]  and the gamma common gamma c chain, this lymphocyte receptor system is critical for T lineage but not for B lineage development.  Ref: 11023514 Blood, 2000
- The IL 7  [ **IL7** ]  receptor is comprised of two chains, IL 7R alpha  [ **IL7R** ]  and gamma c.  Ref: 10379911 Cytokine Growth Factor Rev, 1999
- IL 7R alpha  [ **IL7R** ]  cells harvested from TRAF6 embryos expressed LTalphabeta in response to IL 7  [ **IL7** ]  but not RANKL, demonstrating that the RANK TRAF6 signaling pathway regulates LT alpha beta expression in LN but not in PP.  Ref: 12479827 Immunity, 2002
- Thus it is shown that IL 7R alpha  [ **IL7R** ]  is a component of both the IL 7  [ **IL7** ]  and TSLP receptors, which helps to explain why deletion of the gene that encodes IL 7R alpha  [ **IL7R** ]  affects the lymphoid system more severely than deletion of the gene encoding IL 7  [ **IL7** ]  does.  Ref: 10881176 Nat Immunol, 2000
- Soluble IL 7  [ **IL7** ]  administered to TRAF6 embryos was sufficient to restore LN genesis indicating the functional similarities of the IL 7R alpha  [ **IL7R** ]  inducer cells for LN and PP genesis.  Ref: 12479827 Immunity, 2002
- Taken together, these results suggest that IL 7  [ **IL7** ]  and its receptor complex are essential for 1 up regulating the expression of TdT and IL 7R alpha  [ **IL7R** ] , 2 initiating the production of cmu and 3 promoting the formation of a functional pre BCR in on pro B cells.  Ref: 10657646 J Immunol, 2000

**Non-interactive relationship** (e.g. studied together, co-existance, homology, etc.)

- Using mixed human murine FTOC, we showed a TCR alpha beta, TCR gamma delta lymphocytes, NK cells, and dendritic cells complete their differentiation, b IL 7R alpha  [ **IL7R** ]  signaling and IL 7  [ **IL7** ]  are essential, c a detailed phenotypic and functional analysis of discrete successive steps of positively selected thymocytes, d an efficient transduction of genes in HSC with persistent gene expression throughout the T lymphocyte differentiation, and e adaptation to submerging high oxygen culture increases the test sensitivity to a clonal assay.  Ref: 11268400 Ann N Y Acad Sci, 2000
- We have investigated the interleukin 7  [ **IL7** ]  receptor IL 7R alpha  [ **IL7R** ]  chain gene as a positional and functional candidate gene for susceptibility to multiple sclerosis MS, in view of its chromosomal location on 5p14 p12, a region that has shown suggestive linkage in MS genome screens, and its role in T and B cell proliferation and reactivity.  Ref: 12825072 Eur J Hum Genet, 2003
- Mouse mutants lacking expression of the IL 7  [ **IL7** ]  receptor IL 7R alpha  [ **IL7R** ]  chain are defective in thymopoiesis.  Ref: 9645367 Eur J Immunol, 1998
- In interleukin 7  [ **IL7** ]  receptor IL 7R alpha  [ **IL7R** ]  murine thymocytes, it has been shown that rearrangement of the T cell receptor TCR gamma locus is virtually abrogated.  Ref: 9858510 J Exp Med, 1998
- Proliferation of a factor dependent pre B cell line NAG8 7 in response to either TSLP or IL 7  [ **IL7** ]  was inhibited by anti IL 7R alpha  [ **IL7R** ]  mAbs, suggesting that the functional TSLP receptor complex uses IL 7R alpha  [ **IL7R** ] .  Ref: 9916685 J Immunol, 1999
- Binding of TSLP to the thymic stromal lymphopoietin receptor TSLPR is increased markedly in the presence of the IL 7  [ **IL7** ]  receptor alpha chain IL 7R alpha  [ **IL7R** ] .  Ref: 10881176 Nat Immunol, 2000
- These results demonstrate the increased radiosensitivity of intestinal stem cells within the crypts in IL 7R alpha  [ **IL7R** ]  mice and a role for IL 7  [ **IL7** ]  in the protection of radiation induced apoptosis in these same cells.  Ref: 11207240 J Immunol, 2001
- In response to IL 7  [ **IL7** ]  stimulation, Jak 3 phosphorylation was markedly reduced in both patient cells as well as in COS cells reconstituted with mutant IL 7R alpha  [ **IL7R** ] .  Ref: 11023514 Blood, 2000
- Moreover, reverse transcriptase polymerase chain reaction and fluorescence activated cell sorter analysis revealed that dermis infiltrating lymphocytes expressed various cytokines including IL 4 and IL 7  [ **IL7** ] , and several activation markers for T cells CD44, CD69, IL 2R alpha, in addition to IL 7R alpha  [ **IL7R** ] .  Ref: 9579538 J Invest Dermatol, 1998
- Breeding experiments with IL6 and IL 7r alpha  [ **IL7R** ]  deficient mice, indicated that induction of this extrathymic pathway by the OM transgene occurred in the absence of IL 6, but was strictly dependent on IL 7  [ **IL7** ]  receptor signaling.  Ref: 10210329 Exp Hematol, 1999
- An unexpected finding was noted in allogeneic bone marrow transplant studies using IL 7  [ **IL7** ]  receptor null IL 7R alpha  [ **IL7R** ]  mice as recipients.  Ref: 11207240 J Immunol, 2001
- Murine pro B cells require IL 7  [ **IL7** ]  and its receptor complex to up regulate IL 7R alpha  [ **IL7R** ] , terminal deoxynucleotidyltransferase, and c mu expression.  Ref: 10657646 J Immunol, 2000
- Expression of lymphotoxin LT alpha and LTbeta in IL 7  [ **IL7** ]  receptor IL 7R alpha  [ **IL7R** ]  cells in the intestine indicates that defects of Il7ra and Lta mice are due to functional inability of IL 7Ralpha cells in the induction of PP anlage.  Ref: 10330270 Int Immunol, 1999
- Given diminished T cell development in  **Il7**  or  **Il7r**  deficient mice.  Ref: 9843216 Nat Genet, 1998
- Substitution of proline with serine in the extracellular portion of IL 7R alpha  [ **IL7R** ]  did not affect IL 7R alpha  [ **IL7R** ]  messenger RNA mRNA and protein expression, but severely compromised affinity to IL 7  [ **IL7** ] , resulting in defective signal transduction.  Ref: 11023514 Blood, 2000
